# The Young PI Buzz: Learning from the Organizers of the Junior Principal Investigator Meeting at ISMB-ECCB 2013

**DOI:** 10.1371/journal.pcbi.1003350

**Published:** 2013-11-07

**Authors:** Jeroen de Ridder, Yana Bromberg, Magali Michaut, Venkata P. Satagopam, Manuel Corpas, Geoff Macintyre, Theodore Alexandrov

**Affiliations:** 1 Delft Bioinformatics Lab, Faculty of Electrical Engineering, Mathematics and Computer Science, Delft University of Technology, Delft, The Netherlands; 2 Department of Biochemistry and Microbiology, Rutgers University, New Brunswick, New Jersey, United States of America; 3 Department of Genetics, Rutgers University, Piscataway, New Jersey, United States of America; 4 Computational Cancer Biology, Netherlands Cancer Institute, Amsterdam, The Netherlands; 5 Luxembourg Centre for Systems Biomedicine (LCSB), University of Luxembourg, Campus Belval, House of Biomedicine, Esch-sur-Alzette, Luxembourg; 6 The Genome Analysis Centre, Norwich Research Park, Norwich, United Kingdom; 7 NICTA Victorian Research Laboratory, The University of Melbourne, Melbourne, Australia; 8 Departments of Biochemistry and Mathematics, University of Bremen, Bremen, Germany; 9 SCiLS GmbH, Bremen, Germany; 10 Steinbeis Innovation Center SCiLS Research, Bremen, Germany; 11 Skaggs School of Pharmacy and Pharmaceutical Science, University of California San Diego, La Jolla, California, United States of America

## Introduction

If you are a young principal investigator (PI) in the field of computational biology or bioinformatics, you may have noticed recently there is a buzz surrounding you: a plethora of meetings and seminars are being organized specifically for young PIs (P2P workshop at ISMB 2012, An Excellent Research Career Workshop 2012, EMBO Young Scientists' Forum, Young PI Forum at Weizmann Institute 2009–2013, Young Investigators' Meeting by NCI). The challenges faced by young PIs are being discussed widely [1], particularly across social media [2]; funding agencies are searching for new ways to encourage young investigators; new awards are being created; and novel journals, such as EuPA Open Proteomics, provide opportunities tailored for junior scientists. Picking up on this buzz and recognizing the need for a discussion platform, PLOS has established the About My Lab collection of publications. This article is a part of this collection, highlighting the latest event organized by, and for, young PIs: the Junior PI (JPI) meeting.

The JPI meeting took place in Berlin, Germany, at this year's ISMB-ECCB 2013, the flagship conference of the International Society for Computational Biology (ISCB). With the support of the ISCB Board of Directors, the meeting was conceived and organized by a group of ISCB's young PIs, most of whom are former ISCB Student Council leaders. The meeting was a mixture of scientific talks, round-table discussions, and peer-to-peer interaction. To facilitate discussion and interaction, all participants introduced themselves during the joint breakfast. This was followed by three Frontiers in Science talks, in which researchers who recently started their own group gave a review-like overview of their research field and the challenges ahead. The keynote, by Jean Peccoud, dealt with how to run a research lab as a business [3], and how to use tracking tools to account for the productivity of lab members, which invoked plenty of discussion. In the afternoon, several round-table discussions ensued, with summaries presented to the entire audience at the end of the meeting. Since the prospective participants were asked in advance for topics of importance, these discussions were precisely tailored to reflect the interests of the audience.

The meeting turned out to strike the right balance between scientific talks, experience exchange, getting to know each other, and networking opportunities. The success of the JPI meeting, while critically dependent on the input of the participants, may also be accredited to its organizers, each of whom brought his/her own experience, questions, and passions. Interestingly, some of the organizers are still in the postdoc-PI transition phase, which may explain why they are highly motivated to improve the life of a young PI. Moreover, it is becoming increasingly common in modern science for many postdocs to be involved in supervision of research staff, blurring the conventional distinction between a postdoc and PI. This rise of the postdoc as principal investigator was reflected in a recent report by the European University Institute [4].

This article is different from other About My Lab articles, each following the approach “one author—one interview.” Inspired by the experimental approach of the JPI meeting itself, we present you with six short interviews with the JPI meeting organizers, carried out by the Guest Editor of About My Lab (TA). By providing different opinions, these interviews shed light on some of the key issues of a young PI's career.

## Interview 1. Why Junior PIs Should Learn to Say No and Take Time to Reflect

*Jeroen de Ridder, Assistant Professor in the Delft Bioinformatics Lab, Delft University of Technology. Two and a half years into the five-year tenure track. Former chair of the ISCB Student Council. Co-founder of RSG-Netherlands. Supervising two PhD students and four MSc students.*

The first advice I received from my university-appointed mentor, virtually while still shaking hands, was: “You will need to learn to say no.” One year into my tenure track, I knew exactly why that was very valuable advice. Until then, I felt a little bit like the guys from the movie *The Hangover*, who woke up in a penthouse suite of a hotel, after what seemed to be the best party of their lives. Empty bottles everywhere, furniture scattered and destroyed, loose chickens in the living room, a tiger in the bathroom, and a single question in mind: What the hell has happened? In my case, tenure track happened.

*“A tiger in the bathroom, and a single question in mind.”*

While I had not actually been partying all year, I did somehow manage to commit myself to many more weekly lab meetings and committee memberships than I could handle. Between teaching some courses that I took over, plowing through the 200 CVs in my inbox to select a candidate for the open position in my lab, and supervising students—who had grown accustomed to too many hours of weekly interaction—there was practically no time left to do what the tenure track was all about: Set up a lab in which people collectively strive towards excellent science.

Since then, I discovered that in order to say no, it is important to build moments of reflection into your day-to-day activities. I now find myself scheduling afternoons away from the office, for instance, to reflect on which projects are worth pursuing and to devise strategies to keep focused on the science that I really want to do. Moreover, these times are great to reflect on how I can improve my coaching of people, reduce the amount of interaction time with my students, and increase the cohesion and synergy in the group. All in all, these sessions enable me to lead the lab, instead of the lab leading me. As a result, most furniture is back where it should be, although I am still working on getting rid of the tiger—I still have 2.5 years.

## Interview 2. Why Every Young PI Should Organize a Workshop, a Conference, or Both

*Yana Bromberg, Assistant Professor at Department of Microbiology and Biochemistry, adjunct Assistant Professor at Department of Genetics, Rutgers University. Three years into the six-year tenure track. Supervising two postdocs, three PhD students, two undergraduate students, and three high school students.*

Participating in a conference is great. For me, the best part is seeing my research fit perfectly into the “big picture” of science. Organizing a meeting, on the other hand, is a time- and energy-draining black hole. I know this first hand. Since my last year as a postdoc (2010) I've (co-)organized and (co-)chaired four oral and poster ISMB sessions, three SNP-SIGs, two grant-related workshops, and the JPI meeting, not to mention smaller scientific sessions. Make no mistake about it—organizing is hard [5], but it has its perks.

First of all, to organize a great meeting you need to *pick a clear mission*; i.e., decide on the focus and topics of discussion. For example, while inevitably every year the primary topic of the SNP-SIG is genomic variation, we change the yearly focus topics to allow presentation of different perspectives in the field. Second, you should try to *follow your instincts when designing the flow/framework*. Some talks or sessions may be designated as being from different fields, but if your intuition tells you to put them together, then consider it. Finally, you *should communicate with the speakers (beforehand) and the meeting audience*. This gives you the opportunity to meet the field-relevant people—potential collaborators or facilitators/funders of your research, as well as future students or postdocs.

Do it right, and you will feel like you've just had a perfect learning and discovery experience hand-tailored just for you. You'll have met people who are just as passionate about science as you are and, importantly, they have met you. They may also keep you, the up-and-coming new PI, in mind for their next initiative. However, if they invite you to organize again…do think about that time drain.

*“Perfect learning and discovery experience hand-tailored just for you.”*

## Interview 3. The Importance of Building a Professional Network

*Magali Michaut, postdoc at the Computational Cancer Biology group, Netherlands Cancer Institute. Founder of RSG-France and RSG-Europe, former secretary of the ISCB Student Council.*

Is it because I entered the field of computational biology via protein interaction networks, or maybe because I strongly value the support and opinion of people around me? I don't know. What I do know is that I like to build and maintain a good network of connections. Working in France, the UK, Canada, and the Netherlands has enabled me to discover and connect with various communities. In addition, I spend quite some time attending conferences, giving invited lectures, and visiting labs: all great opportunities to meet people and become familiar with their work. Online tools such as LinkedIn are perfect aids to maintain these links with my colleagues. I even have a separate file to keep track of the people I meet. Maybe you are in it!

My network has proven extremely useful. When I was looking for my first postdoc, I asked the colleagues I knew (only a few at the time) to give me suggestions. They turned out to be quite helpful in finding the right position. In my research projects, I enjoy creating collaborations between scientists with different backgrounds, which is essential for the interdisciplinary projects I work on—I simply can't be an expert on everything. I try to connect with the best and build synergistic collaborations that ensure the project can benefit from a wide range of expertise. In other words, I use my colleagues to help me, and, also of importance, I help them in return. As a result, I am often contacted to recommend or establish a connection with collaborators. On such occasions, I do my best to facilitate the interaction, just like a chaperone protein. For me, it is important to be part of the community, as I like to think that we are all part of the same team striving towards furthering science [6]. I also always keep in mind that helping others can be even more rewarding than helping yourself.

*“Helping others can be even more rewarding than helping yourself.”*

## Interview 4. Benefits and Drawbacks of Working on a Big Collaborative Project

*Venkata P. Satagopam, Research Scientist at the Bioinformatics core, Luxembourg Centre for Systems Biomedicine (LCSB), University of Luxembourg. Co-founder of the ISCB Student Council, initiator of ISCB Student Council Internship Program.*

While at EMBL-Heidelberg, I worked for five years on the EU FP6 project TAMAHUD, which involved two academic and three industrial partners. Currently, at the LCSB, I am involved in a bigger EU IMI project, eTRIKS, consisting of 13 pharma/small biotech companies and three academic sites. From these projects, I have learned that being a young researcher working on a big collaborative project can be a very rewarding experience. In particular, working on a big project allows you to take a peek at how the senior PIs are managing the project, and how they deal with upcoming problems and conflicts. Usually, such projects are interdisciplinary, and each partner approaches the scientific problem from a different angle. This allows young scientists to expand their expertise, pick up terminology, and get to know new technologies, all in an environment which is hands-on.

*“To take a peek at how the senior PIs are managing the project.”*

Notwithstanding, there are definite downsides. First of all, the effort spent on communication is high: up to four telephone conferences per week, eight project meetings per year, and lots of travelling. Although this results in a number of high-impact publications, authorship is typically shared between many coauthors, and your name ends up somewhere in the middle. I have learned, though, in such cases, it is worth exploring whether it is possible to publish your work, with all the details about the methods or algorithms, separate from the collaborative publication. Finally, in my experience, patience is bliss because the bigger the scientific problem, the more people are needed and the longer it takes to bear fruit.

## Interview 5. Why You Should Disseminate Research Results through Social Media

*Manuel Corpas, Project Leader at The Genome Analysis Centre (TGAC). Inaugural Chair of the ISCB Student Council.*

For a young PI, your limited weight in the community, irregular presence at conferences, and few publications per year can be limiting factors in disseminating research outcomes and the promoting work carried out in the lab. If used right, social media like blogs, Facebook, or Twitter can become valuable tools to differentiate yourself from other scientists and provide rapid sharing of ideas [7]. I have experienced first-hand how social media can be a powerful way to boost your professional profile [8].

*“Social media can be a powerful way to boost your professional profile.”*

I regularly write about science on my blog, which is the base for my social media strategy. URLs of my blog entries are automatically posted on my Twitter, Facebook, Google+, and LinkedIn accounts. This, together with regular tweets and commenting on other blog posts, promotes a consistent persona on the Web and attracts potential collaborators. Building a blog with hundreds of visits per day and a Twitter account with thousands of followers in computational biology took me about four years, although frustratingly, taking this long to build such an audience can be easily improved as shown by the rapid uptake of followers to my sister's blog on fashion. My YouTube channel hosts videos of some of my presentations at conferences and pleas for funding. Professionally filmed YouTube videos are particularly good at enhancing one's research profile. Journals like F1000Research now allow the linking of YouTube videos and other media with the published article—another reason to stay on top of these technologies, which, if used wisely, may make a difference on the impact of your research. One project in which I have heavily used social media is a crowdfunding initiative to raise funds for sequencing my family's genomes, in return for releasing them on the web under a CC0 (public domain) license. This evolved into what has now become (half-jokingly) the Corpasome [9]. Although requiring additional time and planning, my Web presence has opened many unexpected opportunities for my professional career development; from cold-call invitations to give talks at conferences, to requests from publishers to post a review of their book on my blog.

## Interview 6. A View from Far Away—The Challenges of Collaboration and Hiring in Australia

*Geoff Macintyre, Postdoctoral researcher at NICTA, Victoria, Australia. One and a half years into first postdoc. Former chair of the ISCB Student Council and former president and founder of RSG Australia. Supervising one postdoc, one MSc student, and one research assistant.*

In my time as a postdoc, I have learned that geographical location of a workplace can contribute to the challenges faced by an aspiring young PI. Performing research in Australia, half a world away from Europe and North America, presents a unique set of challenges, especially concerning communication, travelling, and hiring. I find myself carrying out Skype calls with collaborators at ghastly hours of the morning—there is a limited set of “reasonable” times between Melbourne, Boston USA, and Cambridge UK—and the balancing act means that I usually get the bedtime slot. However, I have found that by working flexible hours and learning to avoid feeling guilty when not at the office from 9am-5pm, I can make this work. With flights taking 28 hours to Berlin, 18 to Vancouver, and 30 to New York, it is frustrating that a quick conference trip involves more days of travel than actually spent at the conference, not to mention the cost of flights. But this is why Australian funding budgets generally have up to AU$10,000 per year for travel, and clever use of the quiet (meeting-less) working environment of a plane means that jet lag is the only real pain. While I am not a PI, I have been in the fortunate position of having acquired funds to hire people to work on some of my projects. However, as for most young PIs, attracting a talented individual is a challenge. After a few rounds of advertising, I was surprised to realize that the geographical distance was an additional barrier—Australia is simply “too far away” in most people's minds. Moreover, many high-quality young researchers in Australia cannot resist the allure of the larger European and North American institutes—making recruiting locally difficult. Given these extra hassles, I wondered how Australia can maintain its competitive research position in niche areas such as medical and agricultural research [10]. Then it dawned on me—by being sure to highlight the unique benefits Australia offers for those considering the move. For instance, articulating that the surplus of medical researchers and emerging demand for computational biologists creates a wealth of opportunities for computational biologists wanting to fast-track their career. In addition, Australian institutes offer a competitive postdoc starting salary of approximately US$80,000. Not to mention that Australia also has some of the most livable cities in the world [11]. These points, and the promise of sun-drenched beaches, can be a large enough carrot for high-quality candidates to make the move “downunder.”

*“Australia is simply ‘too far away’ in most people's minds.”*
